# Supplementary material for: Identification of photoperiod-induced specific miRNAs in the adrenal glands of Sunite sheep (Ovis aries)
Source: Front Vet Sci. 2022 Jul 22;9:888207. doi: 10.3389/fvets.2022.888207 (PMC9354845; doi:10.3389/fvets.2022.888207)
Supplement: Supplementary file 1 [file Data_Sheet_1.ZIP › Supplementary file/miRDeep2 file.zip!/miRDeep2 file/Know_Structure/SP42A2/oar-mir-30a.pdf]

miRBase precursor : oar-mir-30a
Total read count : 123848
oar-miR-30a-5p read count : 114853
oar-miR-30a-3p read count : 8994
remaining reads : 1

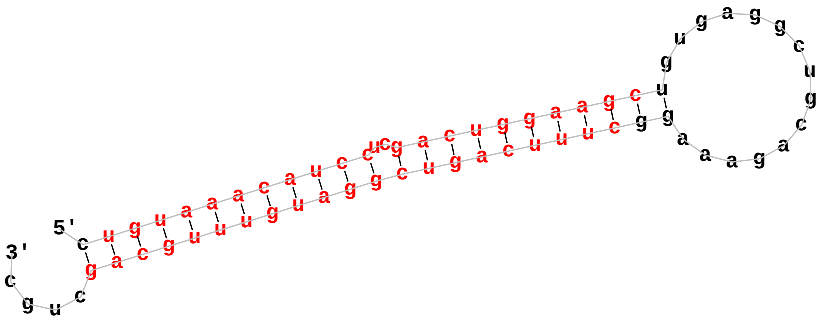

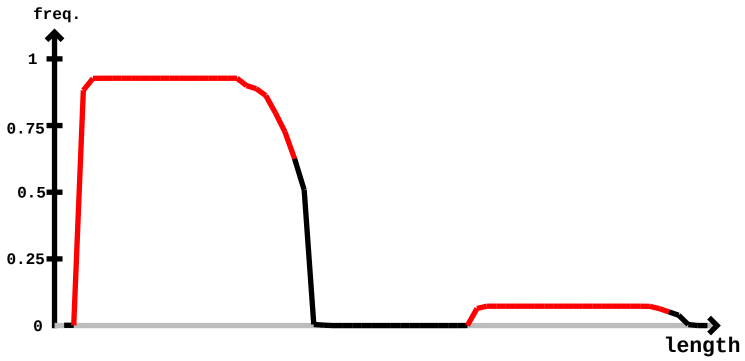


```
                                          oar-miR-30a-3p
  oar-miR-30a-5p
5'- cuguaaacauccucgacuggaagcugugaggcugcagaaaggcuuucagucggauguuugcagcugc   -3'   exp
    ((((((((((((..(((((((((((...............)))))))))))))))))))))))....   reads   mm   sample
    cuguaaacauccuUgacu.................................................   8       1    seq
    cuguaaacauccucgacu.................................................   7       0    seq
    cuguaaacauccucgacug................................................   2       0    seq
    cuguaaacauccuUgacugg...............................................   1       1    seq
    cuguaaacauccucgacugg...............................................   1       0    seq
    cuguaaacauccucgacugga..............................................   21      0    seq
    cuguaaacauccuUgacugga..............................................   1       1    seq
    cuguaaacauccuUgacuggaa.............................................   1       1    seq
    cuguaaacauccucgacuggaa.............................................   10      0    seq
    cuguaaacauccuUgacuggaag............................................   1       1    seq
    cuguaaacauccucgacuggaag............................................   13      0    seq
    cuguaaacauccuUgacuggaagc...........................................   1       1    seq
    cuguaaacauccucgacuggaagc...........................................   27      0    seq
    cuguaaacauccucgacuggaagcu..........................................   23      0    seq
    cuguaaacauccuUgacuggaagcu..........................................   20      1    seq
    .uguaaacauccuUgacu.................................................   3001    1    seq
    .uguaaacauccucgacu.................................................   439     0    seq
    .uguaaacauccucgacug................................................   384     0    seq
    .uguaaacauccuUgacug................................................   990     1    seq
    .uguaaacauccuUgacugg...............................................   1638    1    seq
    .uguaaacauccucgacugg...............................................   1561    0    seq
    .uguaaacauccuUgacugga..............................................   1394    1    seq
    .uguaaacauccucgacugga..............................................   6578    0    seq
    .uguaaacauccucgacuggaa.............................................   7469    0    seq
    .uguaaacauccuUgacuggaa.............................................   1403    1    seq
    .uguaaacauccuUgacuggaag............................................   3850    1    seq
    .uguaaacauccucgacuggaag............................................   8483    0    seq
    .uguaaacauccuUgacuggaagc...........................................   2515    1    seq
    .uguaaacauccucgacuggaagc...........................................   11913   0    seq
    .uguaaacauccuUgacuggaagcu..........................................   33849   1    seq
    .uguaaacauccucgacuggaagcu..........................................   23531   0    seq
    .uguaaacauccuUgacuggaagcug.........................................   57      1    seq
    .uguaaacauccucgacuggaagcug.........................................   14      0    seq
    .uguaaacauccuUgacuggaagcugu........................................   23      1    seq
```

```
                                          oar-miR-30a-3p
 oar-miR-30a-5p
cuguaaacauccucgacuggaagcugugaggcugcagaaaggcuuucagucggauguuugcagcugc

.uguaaacauccucgacuggaagcugu........................................   4     0     seq
..guaaacauccuUgacug................................................   2     1     seq
..guaaacauccuUgacugg...............................................   3     1     seq
..guaaacauccucgacugg...............................................   1     0     seq
..guaaacauccuUgacugga..............................................   17    1     seq
..guaaacauccucgacugga..............................................   4     0     seq
..guaaacauccucgacuggaa.............................................   6     0     seq
..guaaacauccuUgacuggaa.............................................   11    1     seq
..guaaacauccucgacuggaag............................................   10    0     seq
..guaaacauccuUgacuggaag............................................   49    1     seq
..guaaacauccuUgacuggaagc...........................................   49    1     seq
..guaaacauccucgacuggaagc...........................................   36    0     seq
..guaaacauccuUgacuggaagcu..........................................   4708  1     seq
..guaaacauccucgacuggaagcu..........................................   258   0     seq
..guaaacauccuUgacuggaagcug.........................................   234   1     seq
..guaaacauccucgacuggaagcug.........................................   4     0     seq
..guaaacauccuUgacuggaagcugu........................................   161   1     seq
..guaaacauccucgacuggaagcugu........................................   1     0     seq
...uaaacauccuUgacuggaagcu..........................................   23    1     seq
...uaaacauccucgacuggaagcu..........................................   17    0     seq
...uaaacauccucgacuggaagcug.........................................   2     0     seq
...uaaacauccuUgacuggaagcug.........................................   17    1     seq
...uaaacauccuUgacuggaagcugu........................................   6     1     seq
....aaacauccuUgacuggaagcu..........................................   1     1     seq
....................................gaaaggcuuucagucggauguuugcagc...   1     0     seq
........................................Agcuuucagucggauguuu........   1     1     seq
.........................................gcuuucagucggauguuuAc......   4     1     seq
.........................................gcuuucagucggauguuugc......   1     0     seq
.........................................gcuuucagucggauguuuAca.....   19    1     seq
.........................................gcuuucagucggauguuuAcag....   7     1     seq
.........................................gcuuucagucggauguuuAcagc...   4     1     seq
..........................................cuuucagucggauguuu........   18    0     seq
..........................................cuuucagucggauguuug.......   17    0     seq
..........................................cuuucagucggauguuuA.......   23    1     seq
..........................................cuuucagucggauguuuAc......   814   1     seq
..........................................cuuucagucggauguuugc......   181   0     seq
..........................................cuuucagucggauguuuAca.....   1252  1     seq
..........................................cuuucagucggauguuugca.....   191   0     seq
..........................................cuuucagucggauguuugcag....   364   0     seq
..........................................cuuucagucggauguuuAcag....   1090  1     seq
..........................................cuuucagucggauguuugcagc...   1186  0     seq
..........................................cuuucagucggauguuuAcagc...   2681  1     seq
..........................................cuuucagucggauguuugcagcu..   90    0     seq
...........................................uuucagucggauguuuU.......   1     1     seq
...........................................uuucagucggauguuuA.......   1     1     seq
...........................................uuucagucggauguuugc......   25    0     seq
...........................................uuucagucggauguuuAc......   70    1     seq
...........................................uuucagucggauguuugca.....   7     0     seq
...........................................uuucagucggauguuuAca.....   27    1     seq
...........................................uuucagucggauguuuAcag....   41    1     seq
...........................................uuucagucggauguuugcag....   51    0     seq
...........................................uuucagucggauguuugcagc...   225   0     seq
...........................................uuucagucggauguuuAcagc...   223   1     seq
...........................................uuucagucggauguuugcagcu..   367   0     seq
...........................................uuucagucggauguuugcagcug.   11    0     seq
............................................uucagucggauguuuAcagc...   1     1     seq
............................................uucagucggauguuugcagc...   1     0     seq
```
